# One-tube SARS-CoV-2 detection platform based on RT-RPA and CRISPR/Cas12a

**DOI:** 10.1186/s12967-021-02741-5

**Published:** 2021-02-16

**Authors:** Yangyang Sun, Lei Yu, Chengxi Liu, Shanting Ye, Wei Chen, Dechang Li, Weiren Huang

**Affiliations:** 1 Department of Urology, International Cancer Center, Shenzhen Second People’s Hospital, The First Affiliated Hospital of Shenzhen University, Shenzhen University School of Medicine, Shenzhen, 518039 China; 2 International Cancer Center, Shenzhen University School of Medicine, Shenzhen, 518060 China; 3 The First Affiliated Hospital of Shantou University, Shantou, 515041 China; 4 Guangdong Key Laboratory of Systems Biology and Synthetic Biology for Urogenital Tumors, Shenzhen, 518035 China; 5 Shanghai Center for Systems Biomedicine, Key Laboratory of Systems Biomedicine (Ministry of Education), Shanghai Jiao Tong University, Shanghai, 200240 China; 6 Yuebei Second People’s Hospital, Shaoguan, 512000 Guangdong China

**Keywords:** COVID-19, SARS-CoV-2 assay, RT-RPA, CRISPR/Cas

## Abstract

**Background:**

COVID-19 has spread rapidly around the world, affecting a large percentage of the population. When lifting certain mandatory measures for an economic restart, robust surveillance must be established and implemented, with nucleic acid detection for SARS-CoV-2 as an essential component.

**Methods:**

We tried to develop a one-tube detection platform based on RT-RPA (Reverse Transcription and Recombinase Polymerase Isothermal Amplification) and DNA Endonuclease-Targeted CRISPR Trans Reporter (DETECTR) technology, termed OR-DETECTR, to detect SARS-CoV-2. We designed RT-RPA primers of the RdRp and N genes following the SARS-CoV-2 gene sequence. We optimized reaction components so that the detection process could be carried out in one tube. Specificity was demonstrated by detecting nucleic acid samples from pseudoviruses from seven human coronaviruses and Influenza A (H1N1). Clinical samples were used to validate the platform and all results were compared to rRT-PCR. RNA standards and pseudoviruses diluted by different gradients were used to demonstrate the detection limit. Additionally, we have developed a lateral flow assay based on OR-DETECTR for detecting COVID-19.

**Results:**

The OR-DETECTR detection process can be completed in one tube, which takes approximately 50 min. This method can specifically detect SARS-CoV-2 from seven human coronaviruses and Influenza A (H1N1), with a low detection limit of 2.5 copies/µl input (RNA standard) and 1 copy/µl input (pseudovirus). Results of six samples from SARS-CoV-2 patients, eight samples from patients with fever but no SARS-CoV-2 infection, and one mixed sample from 40 negative controls showed that OR-DETECTR is 100% consistent with rRT-PCR. The lateral flow assay based on OR-DETECTR can be used for the detection of COVID-19, and the detection limit is 2.5 copies/µl input.

**Conclusions:**

The OR-DETECTR platform for the detection of COVID-19 is rapid, accurate, tube closed, easy-to-operate, and free of large instruments.

## Background

The outbreak of the novel severe acute respiratory syndrome coronavirus 2 (SARS-CoV-2) has attracted the attention of people all over the world. By August 1, 2020, 17,396,943 cases and 675,060 deaths worldwide had been recorded [1]. In China, for example, although the spread of the epidemic has potentially been contained, there are still sporadic cases of cluster infection or imported coronavirus disease (COVID-19). Coexistence with the novel coronavirus will become a new normality. To ensure orderly production, life, work and study, large-scale, rapid and accurate viral detection is essential [2].

For SARS-CoV-2, laboratories around the world have developed a variety of viral nucleic acid detection platforms, such as sequencing [3, 4], digital PCR [5], and the widely used real-time reverse transcription PCR (rRT-PCR), as recommended by China CDC and the WHO [6, 7]. Generally, the sensitivity of rRT-PCR assays ranges from 3.8 to 10 RNA copies per reaction, with high specificities. These technical platforms require expensive equipment and professional technicians. Additionally, the testing processes take more than 1.5 h [8].

In vitro nucleic acid detection technologies based on clustered regularly interspaced short palindromic repeats (CRISPR)/Cas targeted recognition and promiscuous cleavage activities have been widely reported [9–12]. At present, the method of combining loop-mediated isothermal amplification (LAMP) with CRISPR/Cas12 [13] or RPA with CRISPR/Cas13 [14] has been used to detect SARS-CoV-2. Relatively complex primers must be designed in LAMP amplification, and more components added to the CRISPR/Cas13a testing mix. Since the reactions of isothermal amplification and CRISPR/Cas interfere with each other, the isothermal amplification step is performed first, after which a small amount of amplification product is transferred to the CRISPR/Cas detection system. That might easily produce aerosol contamination in the lab. These rapid platforms have low cost and equipment requirements. The highest sensitivity of these assays ranges from 1 to 4.5 RNA copies per μl [8].

In this study, OR-DETECTR platform was used to detect SARS-CoV-2. This platform might shorten detection time, reduce equipment cost and not produce aerosol contamination.

## Methods

### Oligonucleotides and crRNAs preparation

Primers with a T7 promoter for RPA amplification are compatible with a CRISPR/Cas12a or CRISPR/Cas13a detection system. The primers for RdRp gene amplification were forward primer with T7 promoter 5′-GAAATTAATACGACTCACTATAGGGctaatgagtgtgctcaagtattgagtgaaat-3′ and reverse primer 5′-caaatgttaaaaacactattagcataagcagt-3*′.* The primers for N gene amplification were forward primer with T7 promoter 5′-GAAATTAATACGACTCACTATAGGGcagcagtaggggaacttctcctgctagaatgg-3′ and reverse primer 5′-tggcctttaccagacattttgctctcaagctg-3*′*. The crRNA for RdRp gene (5′-GGGAAUUUCUACUGUUGUAGAUacauauagugaaccgccacaca-3′) and the crRNA for N gene (5′-GGGAAUUUCUACUGUUGUAGAUcugcugcuugacagauuga-3′) were from synthetic oligo fragments. Each oligo fragment contained a T7 promoter which was used as the template for in vitro transcription at 37 °C for 16 h using HiScribe T7 Quick High Yield RNA Synthesis Kit (NEB). RNA was purified using Agencourt RNAClean XP (Beckman Coulter). RNA was quantified by Nanodrop and diluted in nuclease-free water to working concentrations. The crRNAs and ssDNA reporter (5′-6-FAM-TTATTATT-BHQ1-3′ and 5′-6-FAM-TTATTATT-Biotin-3′) were used for DETECTR detection. The crRNAs (5′-GATTTAGACTACCCCAAAAACGAAGGGGACTAAAACgcagcagcaaagcaagagcagcatcac-3′) targeting N gene and ssRNA reporter (5′-6-FAM-UUUUUC-BHQ1-3′) were used for SHERLOCK (Specific High-Sensitivity Enzymatic Reporter UnLOCKing) detection. All these oligonucleotides were synthesized by General Biosystems, Ltd. (Anhui).

### Artificial sample preparation

COVID-19 RNA reference material for limit of detection (LoD) determination was purchased from the Chinese Academy of Metrology. ORF1ab gene segment (14,911–15,910, GenBank No.NC_045512) was 1.1 × 10^6^ copies/μl and N gene was 8.38 × 10^5^ copies/μl. The RNA standard of the RdRp gene was diluted with TE buffer to 6 concentrations of 100 copies/μl, 50 copies/μl, 25 copies/μl, 12.5 copies/μl, 10 copies/μl, 5 copies/μl, and extra 0 copies/μl (blank control). The RNA standard of the N gene was diluted with TE buffer to 7 concentrations of 80 copies/μl, 40 copies/μl, 20 copies/μl, 10 copies/μl, 5 copies/μl, 2.5 copies/μl, 1.25 copies/μl, and extra 0 copies/μl (blank control).

### Pseudoviruses sample preparation

Pseudoviruses from seven coronaviruses including SARS-Cov-2, SARS-CoV, MERS-CoV, hCoV-229E, hCoV-NL63, hCoV-OC43 and hCoV-HKU1 were purchased from Cobioer Biotechnology Company in Nanjing. A pseudovirus of Influenza A (H1N1) was purchased from Genewell Gene Technology Company in Shenzhen. The eight pseudoviruses were diluted from the original concentration to the 200 copies/μl with TE buffer containing 1% TritonX-100. We then used the TE buffer containing 1% TritonX-100 to serially dilute 200 copies/μl of SARS-Cov-2 pseudovirus, 200 copies/μl, 20 copies/μl, 10 copies/μl, 2 copies/μl, 1 copies/μl, 0.2 copies/μl, 0.1 copies/μl and extra 0 copies/μl (blank control). The diluted pseudoviruses were then incubated at 90 °C for 10 min to rupture the virus and release nucleic acid.

### Human clinical sample collection and preparation

Pharyngeal swab samples were collected from 14 fever patients in Shenzhen Second Peoples’ Hospital by the Clinic Diagnosis Laboratory and the nucleic acids of each sample were extracted with the pre-packaged nucleic acid extraction kit (Da’An Gene., Ltd.), according to the manufacturer’s instructions, resulting in 55 μl extracts for each sample.

An additional 40 pharyngeal swab samples from 40 fever patients in Shenzhen Second Peoples’ Hospital were also collected and extracted. rRT-PCR assay employing 5 μl of extract from each sample, were all negative. The remaining 50 μl extract of each sample was combined and 5 μl of the mixture was once again analyzed by rRT-PCR and confirmed to be SARS-CoV-2 negative. The RNA mix was named N50B.

Patients with fever underwent nucleic acid testing for two consecutive days. Patients who tested positive for two consecutive nucleic acid tests were transferred to designated hospitals for treatment. We continued to test patients who had negative tests and no ground glass shadow on chest X-ray to find the cause and determine treatment. The samples used in this study were nucleic acids extracted from patients’ pharyngeal swabs on day one. These patients underwent two rRT-PCR tests and chest X-rays and were evaluated as negative.

### OR-DETECTR assays

The OR-DETECTR platform combines RPA and CRISPR/Cas12a detection. One RPA pellet per tube was resuspended with 29.5 μl rehydration buffer in the TwistAmp basic RPA kit. RT-RPA reactions containing 5 µl of sample extract, 14.75 μl resuspended RPA solution, 1.2 μl of each primer (10 mΜ), 0.75 μl RNA reverse transcriptase (100 U/ml), 0.85 μl nuclease-free water and 1.25 μl MgAc (280 mM), with the total volume of 25 μl. The multiple RT-RPA reaction contains four primers, each of which is 0.6 μl (10 mΜ). After being gently mixed and centrifuged, the mixture was placed at the bottom of a centrifuge tube. The CRISPR/Cas12a reaction mix consisted of 2 μl NEB Buffer 3.1 (10 ×), 2 μl LbCas12a (100 ng/μl), 2 μl crRNA (200 nM), 2 μl ssDNA reporter, and 12 μl nuclease-free water. This 20 μl CRISPR/Cas12a mix was added to the lid of the centrifuge tube before sealing. The tube was placed in a thermocycler or water bath at 42 °C and incubated for 30 min. The CRISPR/Cas12a mixture in the cap was combined with the RPA reaction product with a short spin. If ssDNA reporter was 5′-6-FAM-TTATTATT-BHQ1-3′, the centrifuge tube was then placed in the PE microplate reader at 42 °C, with the fluorescence signal collected every min for 20 min total. If ssDNA reporter was 5′-6-FAM-TTATTATT-Biotin-3′, the centrifuge tube was then placed in a thermocycler or water bath at 42 °C. The reaction product (3 μl) was diluted in 400 μl water, after which 80 μl was added to the sample hole of the test card (GenDx Suzhou).

### OR-SHERLOCK assay

The OR-SHERLOCK platform is a one-tube detection platform based on RT-RPA and SHERLOCK, termed OR-SHERLOCK. The platform combines RT-RPA and CRISPR/Cas13a detection. The RPA amplification step is the same as for the OR-DETECTR. The CRISPR/Cas13a reaction mix consisted of 2 μl Buffer (400 mM Tris, pH 7.4), 2 μl LwCas13a (20 ng/μl), 1 μl crRNA (10 nM), 2 μl ssRNA reporter (4 μM), 0.5 μl RNAse inhibitor (40 U/μl), 0.1 μl T7 Polymerase (100 U/μl), 0.8 μl rNTP (100 mM each), 1 μl MgCl_2_ (120 mM), and 10.6 μl nuclease-free water. The centrifuge tube was incubated in a PCR machine or water bath at 42 °C for 30 min. The detection process of OR-SHERLOCK was the same as OR-DETECTR.

### rRT-PCR assays

Real-time RT-PCR reaction kit was supplied by Biogerm, which uses the primers recommended by the China CDC. Reactions were conducted in a 25 μl volume following the kit instructions. Reaction cycle parameters were set as reverse transcription at 50 °C for 10 min, denaturation at 95 °C for 5 min, followed by 40 cycles of amplification, 95 °C for 10 s and 55 °C for 40 s.

### Statistical analysis

Statistical analyses were performed using GraphPad Prism. P < 0.001 was considered to indicate a statistically significant difference.

## Results

### The workflow of the OR-DETECTR platform

**Fig. 1:**
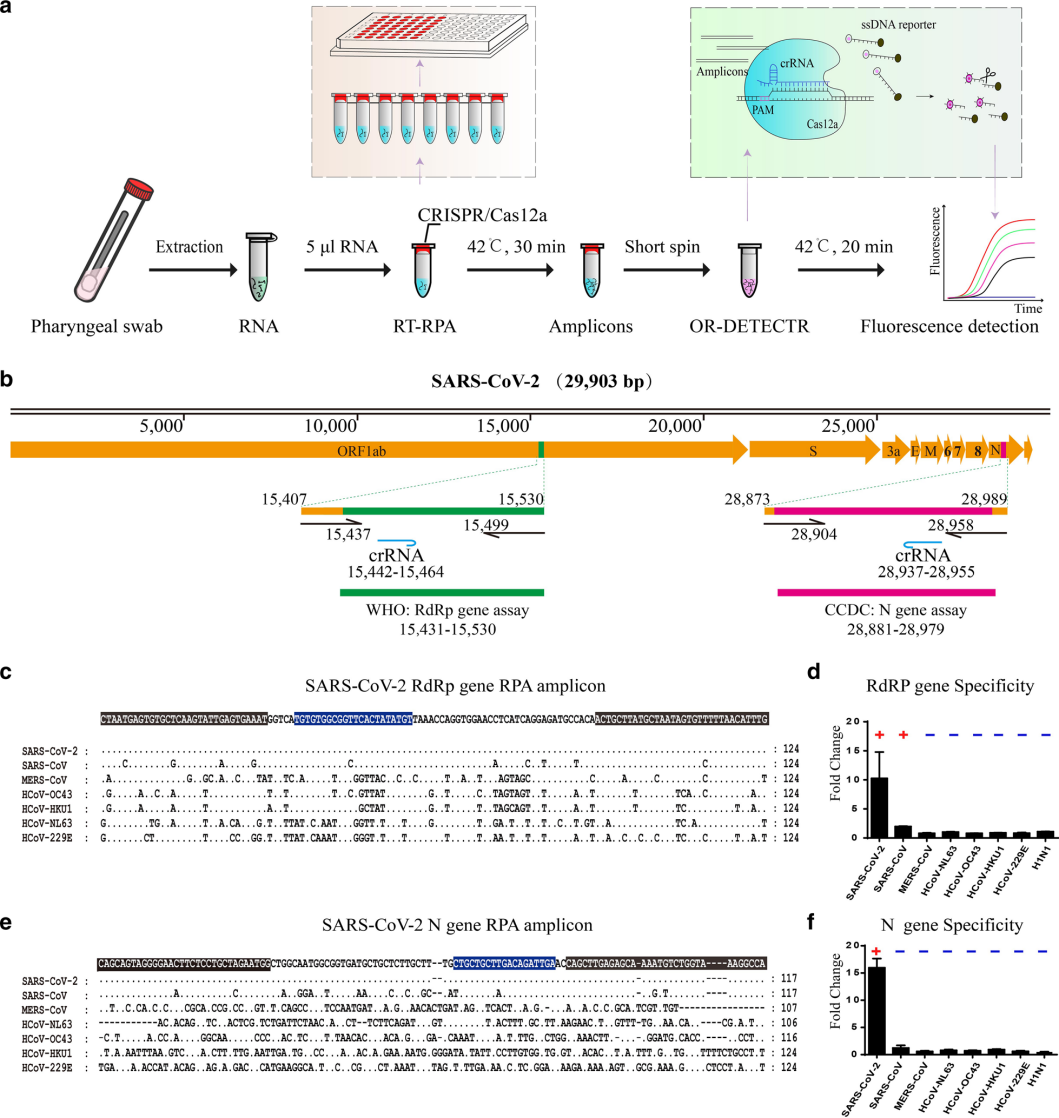
An OR-DETECTR platform for detection of SARS-CoV-2. **a** Schematic of OR-DETECTR testing SARS-CoV-2 workflow. RNA extraction from pharyngeal swab can be used as an input to OR-DETECTR (One-tube detection platform combined RT-RPA based preamplification and CRISPR/Cas12a based DETECTR), which is visualized by a fluorescent reader. The RT-RPA mix is located at the bottom of the centrifuge tube, while the DETECTR mix is located in the transparent tube lid. **b** Genome map showing primers and crRNAs. RT-RPA primers are indicated by black arrows which partially overlap with rRT-PCR primers of RdRp gene (WHO) or N gene (China CDC). Two crRNAs are indicated by light blue curves. **c**, **d** crRNA specificity. The crRNA targeting RdRp gene is designed to detect SARS-CoV-2 and SARS-CoV, which cause severe respiratory infection presented as fever, cough and dyspnea. **e**, **f** The crRNA targeting N gene was specific for SARS-CoV-2. A positive result requires the detection of the SARS-CoV-2 viral N gene target. Dots represent identical nucleotides compared to the sequence of RPA amplicon, (“–”) means sequence gaps are not covered by oligonucleotides. Black shadows represent primer binding sequences and blue shadows represent crRNA recognition sequences. “+” means positive and “–” means negative. Fold change value was used to normalize the fluorescence signal values. Fold change (FC) = (F (PC)-B (PC)) / (F (NC)-B (NC)), F = fluorescence signal value of 19 min, B = fluorescence signal value of 0 min, PC = positive control, NC = negative control. The result was shown as mean ± SD, N = 3. ***P < 0.01

Here, the OR-DETECTR was used for the detection of RNA extracted from pharyngeal swabs of COVID-19 patients. The detection process of the OR-DETECTR platform is shown in Fig. 1a. The RNA sample extracted from the pharyngeal swab is added to the RT-RPA mix at the bottom of the centrifuge tube while the DETECTR mix is located on the transparent tube lid. To reduce the use of the equipment, the reaction temperature for reverse transcription, amplification and detection is set at 42℃. Reverse transcription of viral RNA and high-sensitivity amplification of specific sequences can be completed simultaneously in 30 min at 42 °C. RT-RPA and DETECTR components are physically separated during this amplification process. Following amplification, the DETECTR mix on the lid is mixed with amplification products by centrifugation. The amplicon can be recognized by specific CRISPR RNA (crRNA) segments, while the reaction activates the cis- and trans-nuclease activities of Cas12a. Cas12a nonspecifically cleaves the reporter sequence (quenched fluorescent ssDNA) and the resulting fluorescence generated by the reaction system is recorded within 20 min at 42 °C. There is no need to open the lid during the detection process. The turnaround time is only 50 min. The Cas13a-based molecular detection platform is named Specific High-Sensitivity Enzymatic Reporter UnLOCKing (SHERLOCK) [12]. We have shown that the one-tube detection platform based on RT-RPA and SHERLOCK, termed OR-SHERLOCK, also works. Preliminary comparison of OR-DETECTR and OR-SHERLOCK revealed that the detection effect was essentially the same, although components of the SHERLOCK mix are more complex (Additional file 1: Figure S1, S2).

### The primers and crRNAs design of the OR-DETECTR platform

SARS-CoV-2 is an enveloped virus that contains a positive-sense single-stranded RNA genome of 29,903 nucleotides [15]. We designed primers targeting the RdRp (RNA-dependent RNA polymerase) and N (nucleoprotein) genes of SARS-CoV-2 (Fig. 1b). There is a partial overlap within the RdRp gene amplification region between OR-DETECTR assay and the World Health Organization (WHO) assay [16]. This RdRp gene of pan beta-CoV was designed as a reference assay [2, 17]. The majority of our N gene amplification region overlaps the China-CDC assay (N gene assay) [6]. Including the required protospacer adjacent motif sites (PAM), we designed Cas12a crRNAs to detect RdRp and N gene amplicons. Additionally, we added a T7 promoter to the 5′ end of the forward primers, so that amplification products could also be detected by the OR-SHERLOCK platform (Additional file 1: Figure S2).

### The specificity of the OR-DETECTR platform

There are seven established coronaviruses that infect humans. Four groups are endemic worldwide, namely alpha coronaviruses 229E and NL63, and beta coronaviruses OC43 and HKU1, all causing the common cold. Novel human pathogens can evolve from animal coronaviruses, zoonotically jumping into humans. Three recent examples are the beta coronaviruses SARS-Cov-2 (which causes COVID-19), SARS-CoV (which causes severe acute respiratory syndrome, or SARS), and MERS-CoV (which causes Middle East Respiratory Syndrome, or MERS) [4]. Partial RdRp and N gene sequences of all seven coronaviruses were aligned to the corresponding sequences of amplicons by ClustalW and GeneDoc (Fig. 1c, e). Influenza A is a group of highly contagious seasonal influenza viruses. It can be seen that the RdRp gene sequence and the N gene sequence of the SARS-CoV virus are most similar to SARS-COV-2. Using seven commercially coronaviruses and Influenza A (H1N1) at 200 copies/μl respectively, the OR-DETECTR can distinguish SARS-CoV-2 with no cross-reactivity from the other six coronavirus strains and H1N1 using the N gene target. It can also distinguish SARS-CoV-2 and SARS-CoV with expected cross-reactivity from the other five coronavirus strains and H1N1 with the RdRp gene target (Fig. 1d, f). We included one positive control (PC: 80 copies per μl input) and one negative control (NC: no template) per trial and recorded the fluorescence signal value every min for 19 min. We defined the fluorescence signal value at 0 min as B and the fluorescence signal value at 19 min as F. In each test, the background-subtracted fluorescence was normalized by the negative control, and the fold change values (FC) were generated as FC = (F (PC) − B (PC))/(F (NC) − B (NC)).

### OR-DETECTR platform test clinical samples

**Fig. 2:**
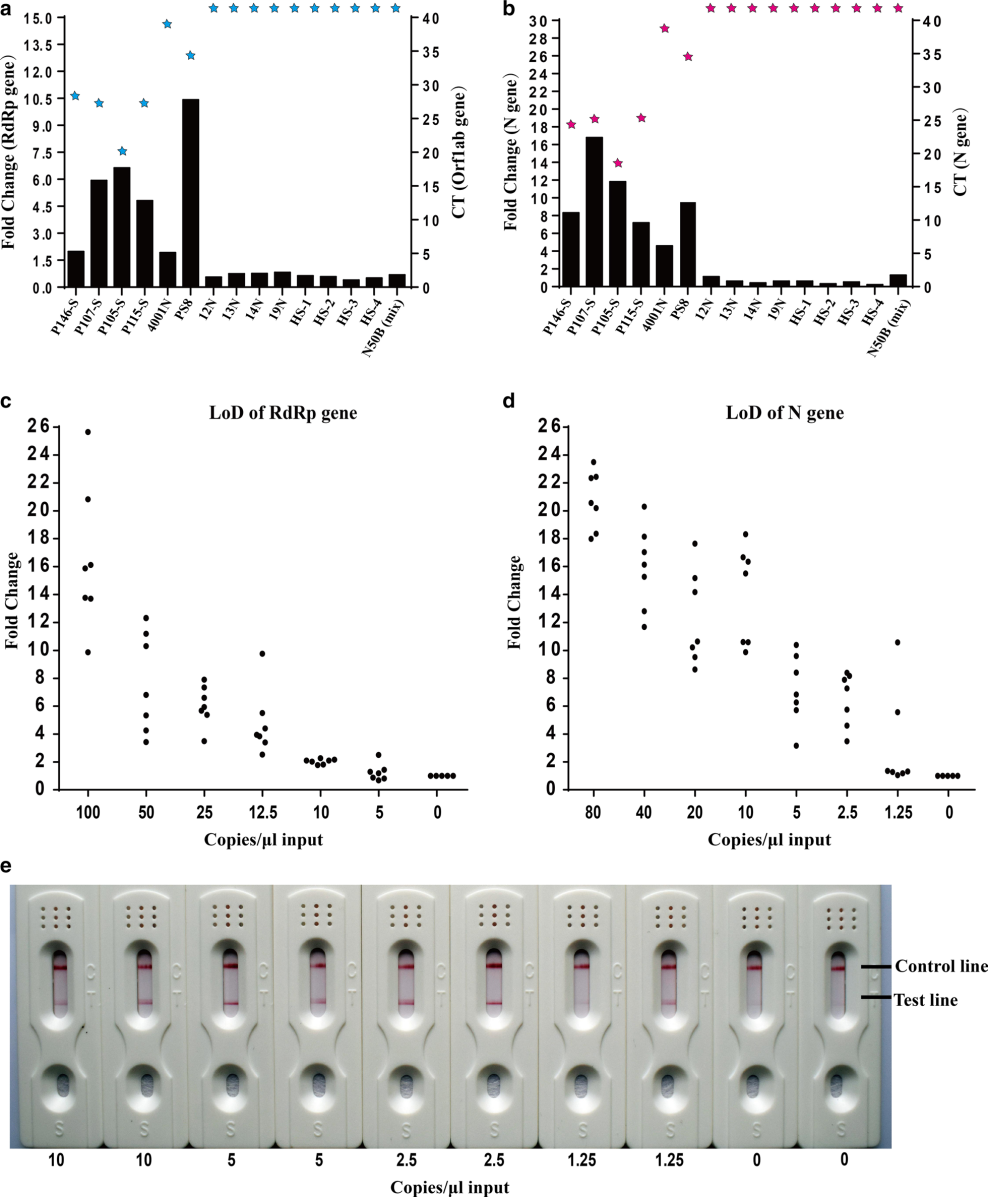
OR-DETECTR assay for SARS-CoV-2 in standard RNA and clinical oropharyngeal swab samples. **a** Fold change data (OR-DETECTR) on RdRp gene and CT values (rRT-PCR) on Orf1ab of patient samples. There were 14 RNA samples from 14 suspected COVID-19 patients. N50S was one RNA mixed sample from 40 fever patients. The CT value of each RNA sample was larger than 40 and undetected. The blue pentagrams (star) represent the CT values of the samples on Orf1ab gene, and the blue pentagrams exceeding the coordinate axis represent the CT values of the samples on Orf1ab gene greater than 40. The black columns represent the FC values of samples on RdRp gene, and the cutoff was 1.6. **b** Fold change data (OR-DETECTR) and CT values (rRT-PCR) on the N gene of suspected COVID-19 patient samples. The samples were the same as a. The red pentagrams (star) represent the CT values of the samples on the N gene, and the red pentagrams exceeding the coordinate axis represent the CT values of the samples on the N gene greater than 40. The black columns represent the FC values samples on the N gene, and the cutoff of the N gene was 2.6. **c**, **d** LoD for OR-DETECTR assay. SARS-CoV-2 RdRp and N gene standard RNA mix were used as the samples for evaluating LoD of OR-DETECTR assay. Standard RNA mix is serially diluted, and there were 7 replicates per dilution. **e** Visual OR-DETECTR assay by test card with lateral flow strip. Standard RNA mix is serially diluted, and there were 2 replicates per dilution

Next, we used the OR-DETECTR platform to test 14 RNA samples extracted from pharyngeal swabs, including six rRT-PCR-positive COVID-19 patients and eight rRT-PCR-negative patients with fever. N50B was a mixture of 40 negative samples, which was included as one sample and also tested via OR-DETECTR. Cycle Threshold (CT) values of N and Orf1ab genes were assessed using the SARS-CoV-2 test kit from BioGerm (Additional file 1: Table S1). We found that 4001 N was a positive sample (FC-RdRp is 1.9 and FC-N is 4.6) by OR-DETECTR, and CT values of 4001 N were 38.9 (Orf1ab) and 36.8 (N gene), meaning this clinical sample had very low viral loads. Although RT-PCR can be used for quantitative analysis and OR-DETECTR cannot, the judgment of negative and positive results between the two platforms was consistent when comparing CT and FC values (Fig. 2a, b, Additional file 1: Fig. S3, Table S1). The negative samples yielded a maximal FC value of 0.8 with the RdRp gene and 1.3 with the N gene, whereas positive samples had a minimal FC value of 1.9 on RdRp and 4.6 on the N gene. Cut-offs (1.6 on RdRp gene and 2.6 on N gene) were set at two times the maximal FC value of negative samples.

### Limit of detection of the OR-DETECTR platform

We next evaluated the limit of detection (LoD) of the OR-DETECTR system with SARS-CoV-2. A commercial RNA standard containing RdRp transcripts (1.1 × 10^6^ copies/μl) and N gene transcripts (8.38 × 10^5^ copies/μl) was serially diluted in TE buffer, with seven replicates at each concentration, and an RNA negative control of buffer alone. The estimated LoD for the OR-DETECTR is 10 copies per µl input with RdRp, as FC values of all seven replicates were greater than 1.6 (Fig. 2c). The LoD with the N gene transcript target is 2.5 copies per µl input, as FC values of all seven replicates were greater than 2.6 (Fig. 2d). The pseudovirus is better for simulating a clinical sample, furthermore, to avoid the use of large-scale equipment and reduce costs, we used the method of heating the lysate to extract the pseudo-viral nucleic acid which had been diluted in gradients. We then evaluated the sensitivity of the OR-DETECTR platform. The LoD for the OR-DETECTR is 20 copies per µl input with RdRp, while the LoD for the N gene transcript target is 1 copy per µl input. The TritonX-100 in the lysate did not affect the nucleic acid detection of the OR-DETECTR platform (Additional file 1: Figure S4). The N gene target has superior sensitivity and specificity to RdRp, meaning the RdRp gene was designated as a reference assay.

### Lateral flow strip detection based on OR-DETECTR

To avoid using large equipment, the OR-DETECTR assay can be visualized by a test card with a lateral flow strip. Activated-Cas12a cleaves FAM-biotin reporter and generates a signal at the first detection line (test line), while uncleaved-reporters are captured at the second detection line (that is, the control line). The OR-DETECTR assay is considered positive if there are two lines, negative if there is one control line, and meaningless if there is no control line. There were two replicates per dilution, and both 2.5 copies per µl input were classified as positive, but only one of 1.25 copies per µl input was positive. The LoD of the lateral flow strip is 2.5 copies per µl input, which is the same as the fluorescence plate reader (Fig. 2e). Additionally, the visual signal was achieved within 4 min by test card. Based on the lateral flow strip, we tried a multiple RT-RPA reaction. The RdRp gene and N gene were amplified in a RT-RPA mixture, after which the amplified product was detected in two CRISPR mixtures containing the crRNA specifically targeting the RdRp or N gene. The results were shown through two lateral flow strips. Results indicated that multiple RT-RPA detection was feasible and that when the sample concentration was 10 copies per µl input, both RdRp and N genes were positive (Additional file 1: Figure S5).

## Discussion

Effective SARS-CoV-2 diagnosis is an important prerequisite for controlling the COVID-19 pandemic. The WHO proposed the ASSURED criteria (Affordable, Sensitive, Specific, User-friendly, Rapid and robust, Equipment-free, and Deliverable to end-users) to determine whether the diagnostic methods being used meet the needs of epidemic control [18]. However, few diagnostic methods meet all standards. For example, rRT-PCR testing is not fast enough, and many kits require qualified laboratories, expensive equipment and professional testing personnel.

We systematically compared the cost, turnaround time, sensitivity, specificity and so on of the current assay with existing COVID-19 diagnostic assays (Table 1).

**Table 1:** Comparison of the rRT-PCR assay and DETECTR (RT–LAMP/Cas12) assay with the OR-DETECTR assay for detection of SARS-CoV-2

	rRT-PCR	DETECTR [13]	OR-DETECTR
LoD (sensitivity)	1 copy/μl input 3.2 copies/μl input [19]	10 copies/μl input	2.5 copies/μl input (RNA standard) 1 copy/μl input (Pseudovirus)
Cross-react (specificity)	No	No	No
Amplification method	PCR	LAMP	RPA
Difficulty of primer design	Easy	Difficult	Easy
Turnaround time	120 min	40 min	50 min
One or more tubes	One	More	One
Temperature	Constant change	62–37 °C	42 °C
Bulky instrumentation required	Yes	Yes/No	Yes/No
Detection of target	N1/N2	E/N	RdRp/N
Results	Quantitative	Qualitative	Qualitative
Cost (each person)	Around ¥20	Around ¥20	Around ¥20

Although rRT-PCR is currently the most commonly used diagnostic method for COVID-19, the LoD of those kits developed by different companies ranges from 100 copies/ml to 1000 copies/ml [8]. A real-time PCR system is a relatively complex, expensive instrument which not all hospitals could configure in their laboratory departments. This means that samples must be sent to the local CDC for testing, increasing testing time as well as various other risks. The detection method based on rRT-PCR is not flexible, since one instrument detects one 96-well plate at a time and the turnaround time is greater than 1.5 h. In contrast, the detection method based on isothermal amplification is flexible, greatly improves detection efficiency as the number of samples ranges from 1 to 384 [13], and the fluorescence detection time is less than 30 min at a time.

The detection methods based on CRISPR/Cas are widely used for detecting COVID-19, using crRNA combined with Cas12 or Cas13 to specifically target DNA or RNA to stimulate the trans-cleavage function of the Cas protein and so amplify the fluorescent signal of the reporter group to detect SARS-CoV-2 nucleic acid amplification products [14, 20, 21]. The amplification of viral nucleic acid is the key to whether it can be specifically detected. Comparative analysis of several isothermal amplification methods indicates that the primer design of the LAMP method is relatively complicated and that it is hard to perform multiple LAMP amplifications [22]. Abbott ID Now, which is based on NEAR (Nicking Enzyme Amplification Reaction), requires only 5 min for a positive result. However, issues with false negativity have been raised due to its relatively high LoD. RT-RPA amplification has high sensitivity, and its primers are easily designed. Therefore, in our OR-DETECTR assay, we used RT-RPA as a potential means of template amplification. In this way, multiple RT-RPA can be performed.

We have developed two detection platforms, OR-DETECTR and OR-SHERLOCK. Through comparison, we found that: (1) the turnaround time, sensitivity, and specificity of OR-DETECTR based on Cas12a and OR-SHERLOCK based on Cas13a are approximately the same; (2) The design of crRNA is simple in the OR-SHERLOCK platform as the PAM region (Protospacer Adjacent Motif) is not needed, but since Cas13a targets RNA, the detection mixture must be added to the components necessary for in vitro transcription, increasing the cost; (3) The crRNA used by OR-DETECTR requires a specific PAM region. As the genome of the SARS-CoV-2 virus is 29,903 nucleotides, it is easy to find suitable PAM regions. Additionally, OR-DETECTR targets DNA, meaning no further components are required.

According to current reports, most COVID-19 detection methods based on isothermal amplification and CRISPR/Cas require a two-step reaction. First, the reverse transcription and isothermal amplification are performed, after which a small amount of amplification product is taken for CRISPR/Cas detection. This is because isothermal amplification and the CRISPR detection interfere with each other. The transfer process requires opening the amplification reaction tube, which will cause aerosol pollution [13, 14]. In clinical laboratories, if aerosol contamination occurs, false-positive results will be produced. Our OR-DETECTR test uses a one-tube detection method. We put the CRISPR mixture in the lid of the tube, and RT-RPA mixture in the bottom of the tube. After the RT-RPA reaction is completed, the amplification product is mixed with the CRISPR mixture by transient centrifugation, after which the fluorescence signal is detected. This reduces the operation of pipetting, shortens the time, and reduces the waste of consumables. Importantly, this can effectively avoid the unavailability of clinical laboratories caused by aerosol contamination.

Lateral flow paper has been applied to CRISPR-based detection methods for signal output. For single-sample detection, this is a good solution as no equipment is required to read the results, and it is promising for COVID-19 detection at home. According to current reports, its LoD is much lower than fluorescence-based detection methods [13]. Our OR-DETECTR assay can be easily read by a fluorescence plate reader to achieve sensitive and high-throughput SARS-CoV-2 detection. Additionally, the sensitivity of our lateral flow paper detection is the same as the fluorescence signal; that is, 2.5 copies per μl input. Finally, our OR-DETECTR test results are consistent with the rRT-PCR test results in the clinic, even for samples with very low viral loads. OR-DETECTR is suitable for clinical laboratories, point-of-care settings with the appropriate equipment, and perhaps for household purposes. Our OR-DETECTR is designed to provide a clear positive or negative result, rather than a quantitative viral load. Considering that the diagnosis of COVID-19 requires a fast, specific, and sensitive method, we do not think this is a disadvantage. Further, our OR-DETECTR method continues to use samples after nucleic acid extraction. Since it is currently difficult to obtain clinical samples, we have no way to continue developing methods for direct nucleic acid amplification from nasopharyngeal swabs.

## Conclusion

Expensive equipment and longer detection time are limitations of the rRT-PCR platform. The currently available CRISPR/Cas-based detection platform requires separation of the amplification and detection steps, increasing the complexity of the operation and the risk of aerosol contamination. Here, we combined RT-RPA with CRISPR/Cas12a DETECTR technology to develop a one-tube, rapid (50 min) test for detection of SARS-CoV-2 in clinical samples, called OR-DETECTR. It can specifically detect SARS-CoV-2 from seven human coronaviruses and Influenza A with LoD of 1 copies/µl input. Results of OR-DETECTR are 100% consistent with rRT-PCR. The enzymes and detection reagents used in the OR-DETECTR detection platform are different from rRT-PCR, which can be used as a complementary detection method to avoid the testing debacle caused by an insufficient supply of reagents in the rRT-PCR platform. With the number of COVID-19 tests required continuing to climb, a high-throughput nucleic acid testing platform is urgently needed. OR-DETECTR is not limited by high-end equipment. It can be used for high-throughput clinical testing or combined with lateral flow strips for individual testing. After the current epidemic situation is controlled, this type of detection platform, with minimal equipment and a short turnaround time, can be widely used in local emergency departments, clinics, airports, stations and other field locations.

## Supplementary Information

**Additional file 1:** Supporting information

## Data Availability

The datasets used and/or analyzed during the current study are available from the corresponding author on reasonable request.

## Abbreviations

CRISPR: Clustered regularly interspaced short palindromic repeats
DETECTR: DNA endonuclease-targeted CRISPR trans reporter
OR-DETECTR: One-tube detection platform based on RT-RPA and DETECTR
COVID-19: Coronavirus disease 2019
SARS-CoV-2: Severe acute respiratory syndrome coronavirus 2
RT-RPA: Reverse transcription and recombinase polymerase isothermal amplification
LAMP: Loop-mediated isothermal amplification
RdRp: RNA-dependent RNA polymerase
N: Nucleoprotein
ORF1ab: Open reading frame 1ab
SHERLOCK: Specific high-sensitivity enzymatic reporter UnLOCKing
OR-SHERLOCK: One-tube detection platform based on RT-RPA and SHERLOCK
crRNA: CRISPR RNA
PAM: Protospacer adjacent motif
rRT-PCR: Real-time reverse transcription polymerase chain reaction
CDC: Centers for disease control and prevention
WHO: World Health Organization
LoD: Limit of detection
CT: Cycle threshold
FC: Fold change
