# Supplementary material for: One-tube SARS-CoV-2 detection platform based on RT-RPA and CRISPR/Cas12a
Source: J Transl Med. 2021 Feb 16;19:74. doi: 10.1186/s12967-021-02741-5 (PMC7884969; doi:10.1186/s12967-021-02741-5)
Supplement: Supplementary file 1 — Additional file 1: Supporting information [file 12967_2021_2741_MOESM1_ESM.doc]

Additional information for

One-tube SARS-CoV-2 detection platform based on RT-RPA and CRISPR/Cas12a

**This PDF file includes:**

Figures S1, S2, S3, S4, S5

Tables S1,


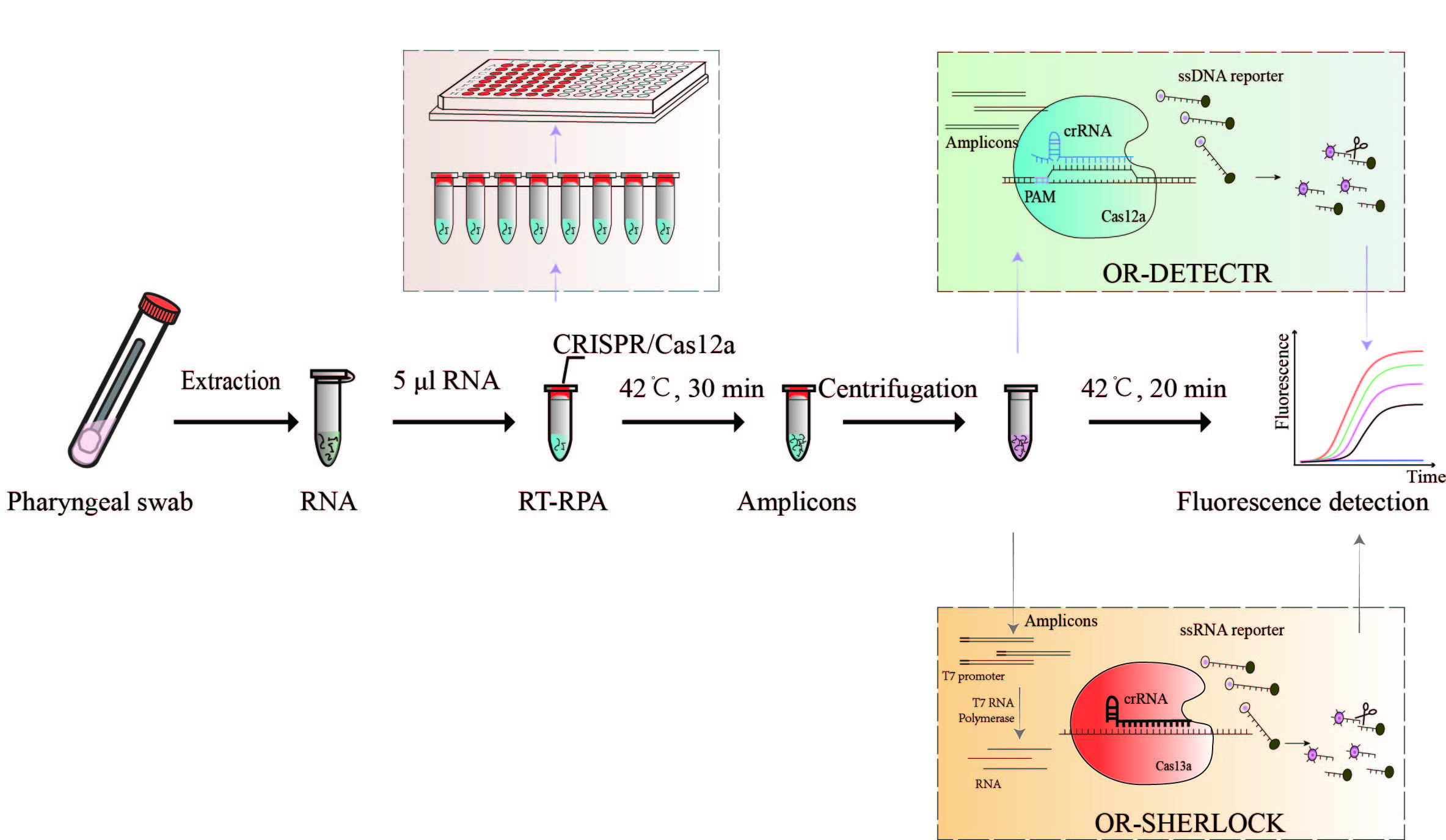


**Figure S1. Schematic of OR-DETECTR and OR-SHERLOCK platform detecting SARS-CoV-2 workflow.** RNA extraction from pharyngeal swab can be used as an input to OR-DETECTR (One-tube detection platform combined RT-RPA based preamplification and CRISPR/Cas12a based DETECTR) or OR-SHERLOCK (One-tube detection platform combined RT-RPA based preamplification and CRISPR/Cas13a based SHERLOCK), which is visualized by a fluorescent reader. The RT-RPA mix is located at the bottom of the centrifuge tube, while the DETECTR mix or the SHERLOCK mix is located in the transparent tube lid.


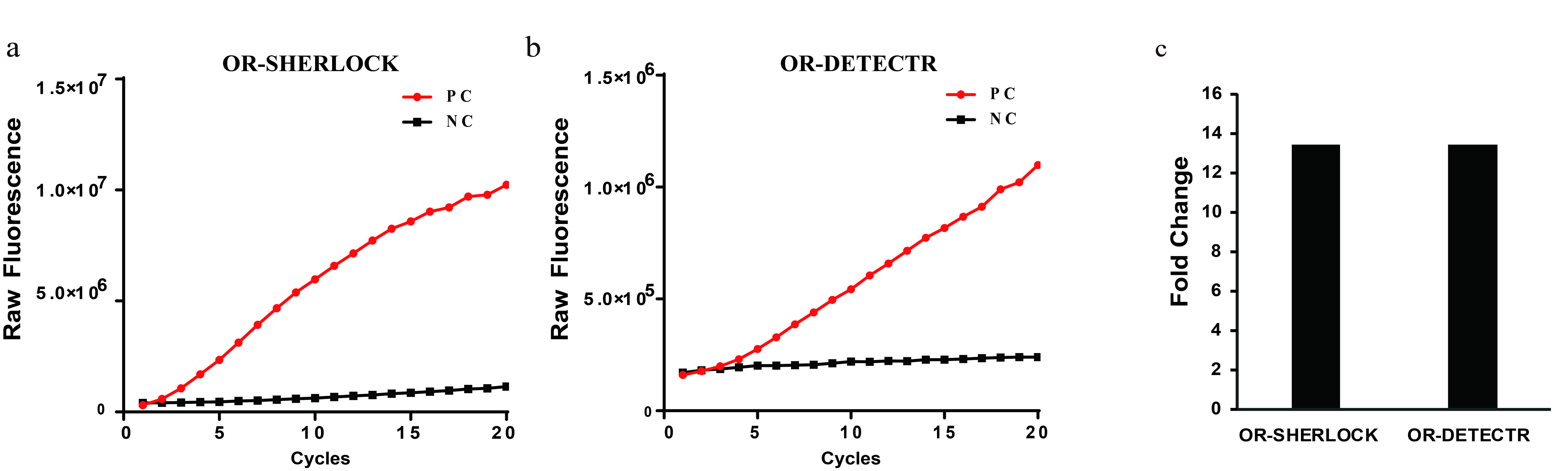


**Figure S2. A preliminary comparison of OR-DETECTR and OR-SHERLOCK.** a, Fluorescent OR-SHERLOCK kinetic curves on N gene (80 copies/μL input). b, Fluorescent OR-DETECTR kinetic curves on N gene (80 copies/μL input). c, Compare the Fold change Values for both DETECTR and SHERLOCK platforms.


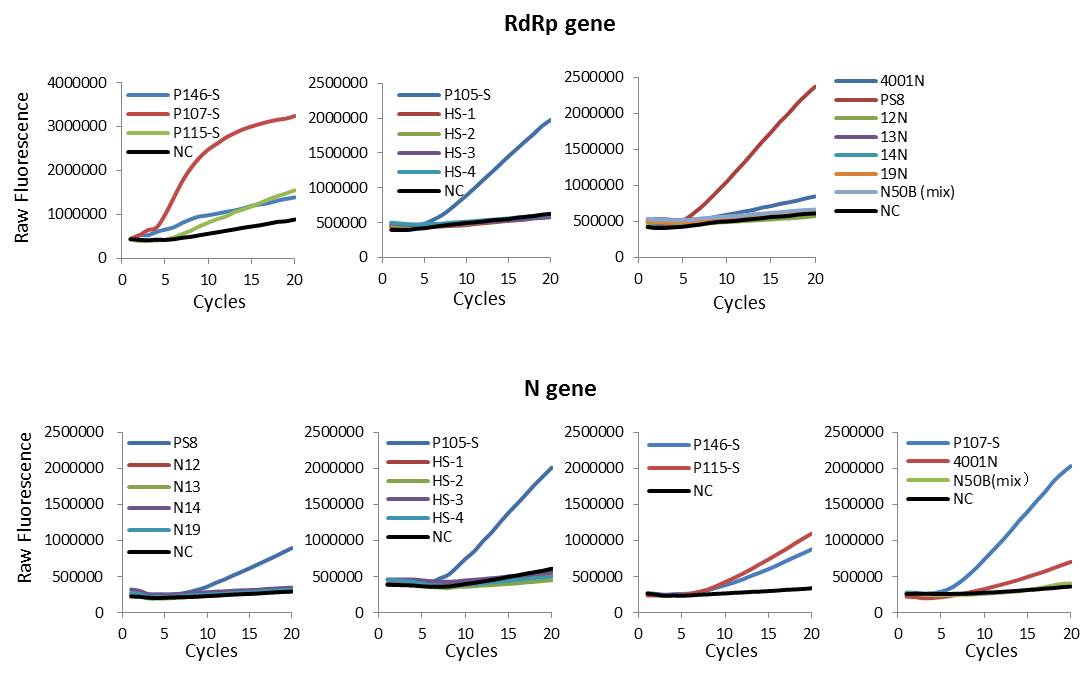


**Figure S3. Fluorescent OR-DETECTR kinetic curves of clinical samples.** 6 COVID-19 infected positive patient samples, 8 COVID-19 infected negative patient samples and a mix sample of 40 negative samples (N50B).


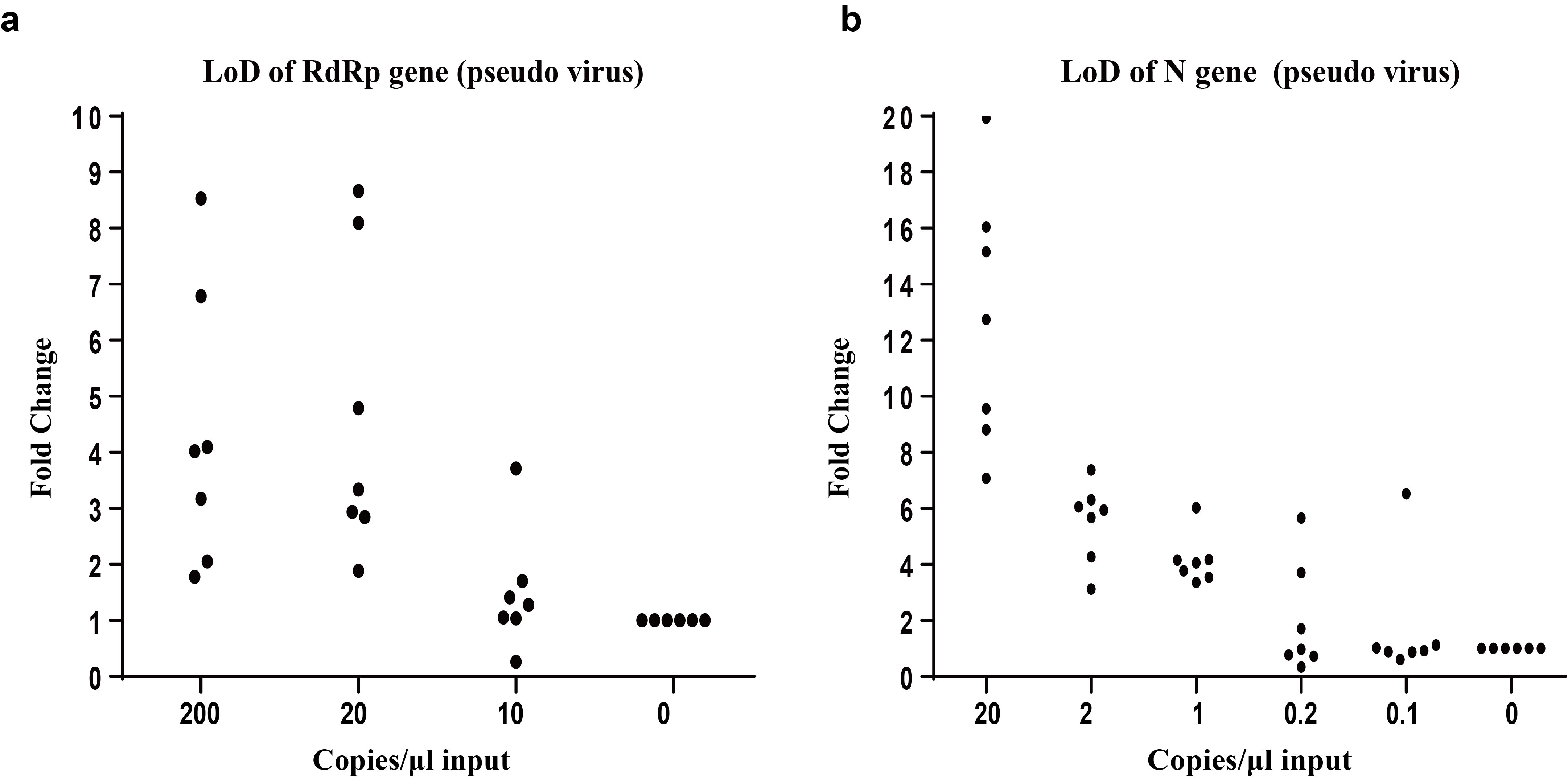


**Figure S4. LoD for OR-DETECTR assay.** a-d,. SARS-CoV-2 pseudovirus was used as the samples for evaluating LoD of OR-DETECTR assay. Pseudovirus sample is serially diluted, and there were 7 replicates per dilution.


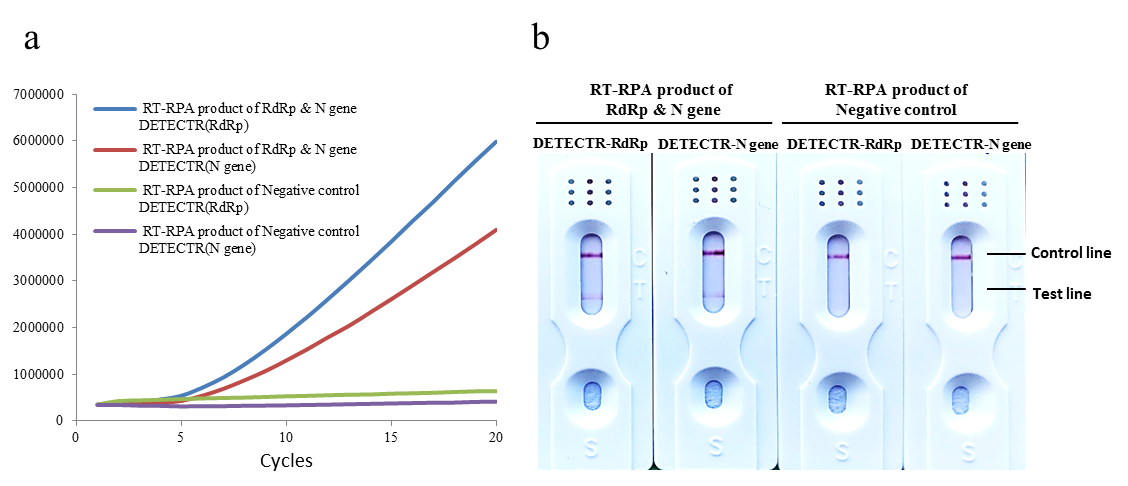


**Figure S5. Signal output of Multiple RT-RPA by fluorescence plate reader and lateral flow strips.** a, Fluorescence plate reader display RdRp and N gene product from multiple RT-RPA. b, Lateral flow strips display RdRp and N gene product from multiple RT-RPA.

Table S1. CT values and FC values of clinical samples

|  | CT value | |  | FC value | |
| --- | --- | --- | --- | --- | --- |
| Clinical Sample | CT-Orf1ab | CT-N |  | FC-RdRp | FC-N |
| P146-S | 28.5 | 24.5 |  | 2.0 | 8.3 |
| P107-S | 27.5 | 25.5 |  | 5.9 | 16.8 |
| P105-S | 20.0 | 18.0 |  | 6.6 | 7.2 |
| P115-S | 27.5 | 26.0 |  | 4.8 | 11.8 |
| 4001N | 38.9 | 36.3 |  | 1.9 | 4.6 |
| PS8 | 37.1 | 34.5 |  | 10.4 | 9.5 |
| 12N | UD | UD |  | 0.6 | 1.1 |
| 13N | UD | UD |  | 0.7 | 0.6 |
| 14N | UD | UD |  | 0.8 | 0.4 |
| 19N | UD | UD |  | 0.8 | 0.6 |
| HS-1 | UD | UD |  | 0.6 | 0.6 |
| HS-2 | UD | UD |  | 0.6 | 0.3 |
| HS-3 | UD | UD |  | 0.4 | 0.5 |
| HS-4 | UD | UD |  | 0.5 | 0.2 |
| N50B (mix) | UD | UD |  | 0.7 | 1.3 |

Note: UD, undetected.
